# Combination strategies enhance oncolytic virotherapy

**DOI:** 10.18632/oncotarget.17643

**Published:** 2017-05-06

**Authors:** Gregory K. Friedman, James M. Markert, George Yancey Gillespie

**Affiliations:** Department of Pediatrics, Division of Pediatric Hematology and Oncology, University of Alabama at Birmingham, Birmingham, AL, USA

**Keywords:** oncolytic, virotherapy, HSV, Aurora A kinase, checkpoint proteins

Oncolytic herpes simplex virus (oHSV) therapy offers great promise at targeting difficult-to-treat solid tumors as evidenced by the first FDA approval of an oncolytic virus, modified HSV-1 talimogene laherparepvec (T-VEC; IMLYGIC) to treat advanced melanoma [1]. There are a number of active studies in children and adults using first generation oHSVs that contain deletions in the neurovirulence gene γ_1_34.5 (G207, ClinicalTrials.gov identifier NCT02457845; HSV1716, NCT00931931) or second generation viruses, like T-VEC (NCT02509507; NCT02211131; NCT02658812; NCT03069378; NCT02626000; NCT02819843) that produces granulocyte-macrophage colony-stimulating factor (GM-CSF) or M032 (NCT02062827) that produces interleukin-12 to augment the oncolytic effect of the virus by stimulating a more robust anti-tumor immune response. The ideal balance between viral oncolysis and immune stimulation is unknown yet very consequential. Cytolysis from virus replication exposes previously unexposed cancer antigens to immune effector cells which are attracted to the area by the virus in the context of a danger response and can attack the tumor; however the immune cells can also remove the virus, and thereby limit viral oncolysis.

Researchers have explored combination approaches with oHSVs including various chemotherapeutics and low-dose radiation to maximize both viral oncolysis and the effect of the added therapy. In most combination studies with chemotherapy drugs, apoptosis was increased but viral replication was not enhanced [2-4]. Interestingly, when combined with oHSV, paclitaxel could be dosed 5-fold lower with similar tumor responses as paclitaxel alone suggesting that oHSV may permit lower doses of chemotherapy to be used resulting in less toxicity for patients [3]. While bevacizumab, a monoclonal antibody against vascular endothelial growth factor (VEGF), decreased virus replication, it still synergized with oHSV in part through enhanced antiangiogenesis and modulation of the innate cellular host response [4]. In other studies, low dose-ionizing radiation augmented virus replication through multiple proposed mechanisms, including increased cellular activity due to host cell responses to DNA damage which may in turn complement viral gene deletions and activate HSV-1 promoters (reviewed in [5]). Radiation may also potentiate innate and adaptive immune responses to augment an anti-tumor response.

In a recent issue of Oncotarget, Currier et al. describe the synergistic effects of combining Aurora A Kinase inhibitor alisertib (MLB8237) with HSV1716, a first-generation oHSV currently in phase I clinical trial in children with non-central nervous system solid tumors (NCT00931931), in neuroblastoma and malignant peripheral nerve sheath cell lines [6]. Alisertib selectively binds and inhibits Aurora A kinase which plays important roles in regulating mitosis. This combination approach resulted in three important findings: 1) In addition to the direct cytotoxicity of alisertib and HSV1716, the virus increased the sensitivity of surrounding cells to alisertib through a paracrine effect whereby infected cells secreted signals that killed some uninfected cells. The authors termed this effect Virus-Induced Therapeutic Adjuvant (VITA). 2) Alisertib increased peak virus production and decreased virus clearance *in vivo* thereby prolonging oncolysis possibly by preventing the influx of virus-mediated NK cells that may limit virus replication. 3) Alisertib prevented the increase of monocyte myeloid derived suppressor cells (MDSCs) induced by HSV1716. MDSCs have been shown to be pro-tumorigenic. Taken together, these exciting results demonstrate how a combination strategy may be used to not only improve the effectiveness of oncolytic virotherapy but also increase the efficacy of the combined drug. Further studies are needed to determine if there is one principal mechanism resulting in the increased antitumor effect or if each of these findings (and perhaps others not yet described) combined are responsible for the outcomes. Additionally, the generalizability of these findings to other models will be interesting to investigate.

In addition to maximizing virus replication and the effect of an added agent, a critical challenge remains exploiting the immune system to create a sustained anti-tumor response. The combination of HSV1716 and alisertib removed unfavorable MDSCs which are known to promote pro-tumorigenic M2 macrophages. Additional opportunities exist with adjuvant immunotherapies such as natural killer (NK) cell therapy and checkpoint molecule inhibitors. Recently, researchers demonstrated synergy between bortezomib, a proteasome inhibitor, and oHSV in causing inflammatory necroptotic tumor cell death [7]. This was accompanied by secretion of inflammatory cytokines which activated NK cells and increased sensitization of tumor cells to NK cell immunotherapy. Tumors may evade immune surveillance through the expression of immunosuppressive checkpoint proteins on the surface of tumor cells or in the tumor microenvironment that negatively regulate immune cell function, and inhibitors of these proteins have been developed and have resulted in some dramatic and durable responses in a variety of solid tumors [reviewed in 8]. These agents may be an ideal combination with oHSV since the virus can activate an antitumor immune response, a foreign gene product such as a cytokine that is produced with viral replication can amplify the response, and checkpoint blockade may further intensify and maintain the antitumor immune response. Studies are underway both preclinically and clinically examining various combinations of checkpoint inhibitors and oHSVs. Combination approaches like the one described by Currier et al. likely hold the key to unlocking the full potential of oHSV, and the challenge for researchers moving forward is to determine the ideal combinations to maximize the benefit of oHSV.
